# Effect of Combined Laser Thermal and Shock Wave Effects on the Mechanical and Tribological Properties of Steels

**DOI:** 10.3390/ma17081809

**Published:** 2024-04-15

**Authors:** Anatoly Bragov, Andrey Lomunov, Evgeny Rusin, Gennady Gavrilov, Andrey Kurkin

**Affiliations:** 1 Research Institute of Mechanics, National Research Lobachevsky State University of Nizhny Novgorod, Nizhny Novgorod 603950, Russia; bragov@mech.unn.ru (A.B.); lomunov@mech.unn.ru (A.L.); 2 Institute of Mechanical Engineering Problems, Russian Academy of Science, Nizhny Novgorod 603024, Russia; eerusin@mail.ru; 3 Department of Materials Science, Materials Technology and Heat Treatment of Metals, Nizhny Novgorod State Technical University n.a. R.E. Alekseev, Nizhny Novgorod 603155, Russia; gavrilov1109@mail.ru; 4 Department of Applied Mathematics, Nizhny Novgorod State Technical University n.a. R.E. Alekseev, Nizhny Novgorod 603155, Russia

**Keywords:** wear resistance, shockwave, hardening, laser thermal hardening, carbon steel

## Abstract

Herein, we present the results of an experimental study on the mechanical properties of Fe-C alloys with different carbon contents (0.2, 0.45, and 0.8%) in a wide range of deformation rates (10^−3^–10^3^ s^−1^) and abrasive wear resistance, which underwent combined laser thermal (laser surface hardening—LSH) and laser shock wave (Laser Shock Peening—LSP) processing. The combined treatment modes included a different sequence of exposure to laser thermal and laser-induced shock pulses on the material. The amplitude and duration of laser-induced shock waves were measured using a laser Michelson interferometer. The mechanical properties of steel samples were studied under conditions of uniaxial tension under static loads on a standard universal testing machine, the LR5KPlus, and under dynamic loading, tests were carried out on a specialized experimental complex according to the H. Kolsky method using a split Hopkinson rod. The abrasive wear resistance of hardened surfaces was studied using the Brinell–Haworth method. Studies have shown that the use of a combination of LSH and LSP treatments leads to an increase in both the mechanical properties of steels and abrasive wear resistance compared to traditional laser hardening. It has been established that in the combinations considered, the most effective is laser treatment, in which LSP treatment is applied twice: before and after LSH. Thus, after processing steels using this mode, an increase in the depth of the hardened layer was recorded—by 1.53 times for steel 20, by 1.41 times for steel 45, and by 1.29 times for steel U8—as well as a maximum increase in microhardness values by 22% for steel 20, by 27% for steel 45, and by 13% for U8 steel. The use of this mode made it possible to obtain the maximum strength properties of the studied materials under static and dynamic loading, which is associated with an increase in the volume fraction of the strengthened metal and high microhardness values of the strengthened layer of traditional LSH. The dependences of abrasive wear of the studied steels after various combinations of LSP and LSH impacts were established. It is shown that the greatest wear resistance of the studied steels is observed in the case when the LSH pulse is located between two LSP pulses. In this case, abrasive wear resistance increases by 1.5–2 times compared to traditional LSH.

## 1. Introduction

Reducing metal consumption and increasing the reliability and durability of machine parts and mechanisms are closely related to the quality of metal alloys. The low quality of mass steel grades in some cases does not allow the requirements of designers to be met when creating new machines and mechanisms. Therefore, it is economically feasible not so much to increase the volume of metal pro-duced, as to improve the hardening technologies of alloys. The introduction of new methods of metal processing, an important technique among which is laser processing [1], makes it possible to use alloys in a hardened state after the implementation of promising strengthening thermomechanical technologies [2,3,4].

The modern machine-building industry needs parts and products with improved performance characteristics. Their production by traditional methods often causes significant technological difficulties. Since the possibilities of classical hardening technologies are practically exhausted, the development of new hardening technologies is of particular relevance, a special place among which is occupied by classical laser surface hardening (LSH) [5,6]. Currently, it is used in industry to increase the resistance of structural elements to static and dynamic mechanical influences. In addition, it is actively used to harden the working surfaces of machining tools and other elements of machines and mechanisms that are subject to significant wear [7].

Another method of laser surface hardening is Laser Shock Peening (LSP) [3,8,9,10,11]. The laser impact treatment (LSP) process is a surface treatment method that causes noticeable structural and phase changes in the thin surface layer with a high level of residual compressive stresses, which leads to a significant improvement in the strength, fatigue, and tribological properties of the material [12].

It is known [13] that laser radiation in the Q-switched mode, interacting with the metal surface, heats it up in a few nanoseconds in the irradiation zone to the evaporation temperature of the material (about 10,000 K) for a thickness of about 1 microns. Further absorption of laser radiation leads to the emission of a vapor plasma torch normally to the surface towards the laser radiation, which induces a response in the form of a short mechanical compression pulse propagating deep into the material. Thus, laser-induced shock waves are formed on the surface of the material, characterized by a short duration and high pressure. After LSP treatment, the structure of the material in the near-surface layer is characterized by all the signs of shock wave action with an ultra-high deformation rate: grain crushing, a high dislocation density, and an increased concentration of deformation vacancies [14]. It should be noted that the mechanical effects of laser-induced plasma shock waves prevail during LSP treatment, and there is almost no thermal effect [15].

In some cases, to increase the amplitude of the pressure pulse, the surface of the samples is covered with a thin layer of material opaque to laser radiation (lead, zinc, black paint), and then covered with a dielectric material transparent to laser radiation (water or glass), limiting the plasma formed [16]. The localization of plasma between the target and a dielectric material transparent to laser radiation slows down the expansion of the plasma and increases the shock pressure in proportion to the acoustic impedance of the limiting medium, which leads to an increase in pressure on the surface of the material [8,12,15]. However, in practical LSP material processing technologies, the application of absorbing coatings and limiting media causes certain technical difficulties and negatively affects the productivity of the processing process.

Along with laser technologies (LSH) [17], there are many alternative traditional technologies that have long been successfully mastered in production, which include classical heat treatment [18], electron beam processing [19], and methods of chemical–thermal treatment based on the use of thermal diffusion to introduce metal atoms, such as C, N, or B atoms, into the surface layer of a material in order to change its chemical composition and microstructure. The main methods of chemical–thermal treatment are carburization, boriding, nitriding, and nitrocarburization [18]. It should be noted that classical heat treatment and chemical–thermal treatment are resource-intensive and polluting technologies. These technologies face problems of high energy consumption, low efficiency, and high environmental pollution. Wastewater, waste gases, dust, and sludge generated as a result of complex processes, including heating, cooling, and cleaning, are the main causes of environmental pollution and pose a threat to human health. At the same time, it is necessary to note the obvious advantages of laser processing technologies compared to classical technologies, the most important of which are the locality of impact; energy saving; the possibility of adjusting the radiation energy; a lack of quenching media; the high environmental friendliness of the technology; no need for final grinding of the workpiece surface; laser surface finishing processes are suitable for both single and mass-produced parts; and the ability to automate laser processing and integrate the laser system into the processing complex.

In such a situation, the issue of using laser technologies should be resolved as a result of a comprehensive technical, economic, and environmental analysis. The existing experience and prospects for the industrial development and implementation of laser technologies in industry show that a constant and in-depth comparative study of the processes, equipment, and economics of laser and competing industrial technologies is necessary. The most promising implementation of laser technologies is in industries that require high-quality processing of expensive products (instrument making, mechanical engineering, etc.).

The urgent task of laser technology is to find new ways to improve its efficiency and practical effectiveness. Certain prospects for improving the parameters and technical and economic indicators of laser processing open up when using a combination of laser thermal irradiation (LSH) with the impact of an energy pulse of a different physical nature. It is most simple to use an additional energy source as a pretreatment that modifies either the surface (plasma, thermal, electroplating, ultrasonic exposure) or the internal structure of the material (pre-thermal or plastic treatment). Such measures, according to studies [20,21], contribute to a better susceptibility of laser radiation by the surface of the material, reduce energy consumption, more fully realize the advantages, and mitigate a number of disadvantages inherent in laser radiation used as a heat source. LSP can serve as an active source of additional energy. In the works [22,23], the high efficiency of modifying the structure of steels by LSP exposure was shown to increase the parameters of subsequent LSH.

An important aspect of increasing the durability of a wide class of machine parts is the quality of the metal, and not the entire section of the product, but the structural condition and physical and mechanical properties of the surface layer. It is the surface layer that determines the operational properties of the parts—wear resistance, strength, the resistance of the material to fatigue failure, contact endurance, corrosion resistance, etc. By now, the available opportunities for increasing the wear resistance of surfaces by methods of volumetric heat treatment alone have almost completely exhausted themselves. To increase the durability of parts, the following methods are used to improve the quality of the surface layer: surface plastic deformation, chemical–thermal treatment, finishing plasma hardening, ultrasonic treatment, electric spark alloying, and laser and plasma hardening. The most effective way to reduce the cost of manufacturing parts and improve the quality of machines are surface treatment technologies with concentrated energy flows. Such technologies include the processing proposed in this paper, based on a combination of LSH and LSP.

The purpose of this work was an experimental study on the mechanical properties under static and dynamic loads and the abrasive wear resistance of Fe-C alloys with different carbon contents that underwent surface hardening based on LSH and LSP impacts, as well as their combinations.

## 2. Materials and Specimens

Three grades of Russian Fe-C alloys with different carbon contents (C) were selected for research: steel 20 (0.20% C, Russian GOST Standard 1050), steel 45 (0.45% C, Russian GOST Standard 1050), and steel U8 (0.8% C, Russian GOST Standard 1435). The analogues of these Russian steels are forein steels AISI 1020, AISI 1045, and W107, respectively. The chemical composition of the steels is given in Table 1.

**Figure 1:**
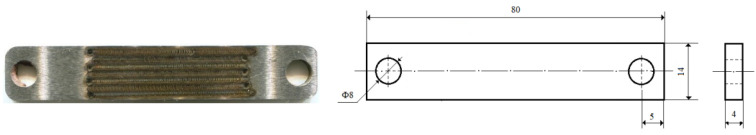
Flat sample after laser treatment for abrasive wear tests.

**Figure 2:**
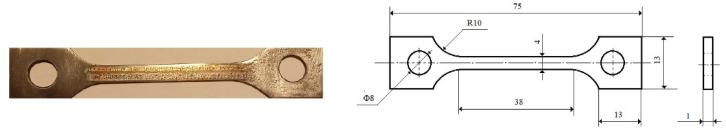
Sample for static tensile tests.

**Figure 3:**
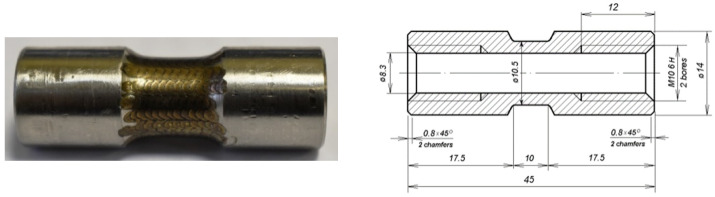
Appearance and configuration of a tubular sample after laser treatment for dynamic tensile tests.

The following test samples were made from these steels:

1. Rectangular plates with dimensions of 40 × 10 mm and a thickness of 4 mm for abrasive wear tests (Figure 1);
2. Blade samples with a working part size of 40 × 4 mm and a thickness of 1 mm for static tensile tests (Figure 2);
3. Tubular thin-walled samples with threaded heads with a working area length of 10 mm and a wall thickness of 1.1 mm for dynamic tensile tests according to the Kolsky method (Figure 3).

The surface of the manufactured samples was ground to Ra = 0.63 microns (Ra is the arithmetic mean deviation of the profile). To obtain a homogeneous structure of the steels and to relieve internal stresses, the samples were subjected to standard furnace full annealing in vacuum. Annealing of samples from steel 20 was carried out at a temperature of 880 °C, from steel 45 at a temperature of 805 °C, and from steel U8 at a temperature of 800 °C. The samples were cooled together with the furnace at a speed of ~100 °C/hour. After annealing, the samples underwent laser treatment. For comparison, some of the samples were not subjected to laser treatment.

Laser surface treatment of rectangular plates for testing for abrasive wear was carried out on one side; for blade samples, treatment was carried out on both sides within the working area, and for tubular samples, along the perimeter and length of the working part of the samples. Flat samples were placed on a two-coordinate desktop. A rotating device with the possibility of horizontal movement was used to process the tubular sample.

## 3. Research Methods

### 3.1. Methods of Combined Laser Processing

**Figure 4:**
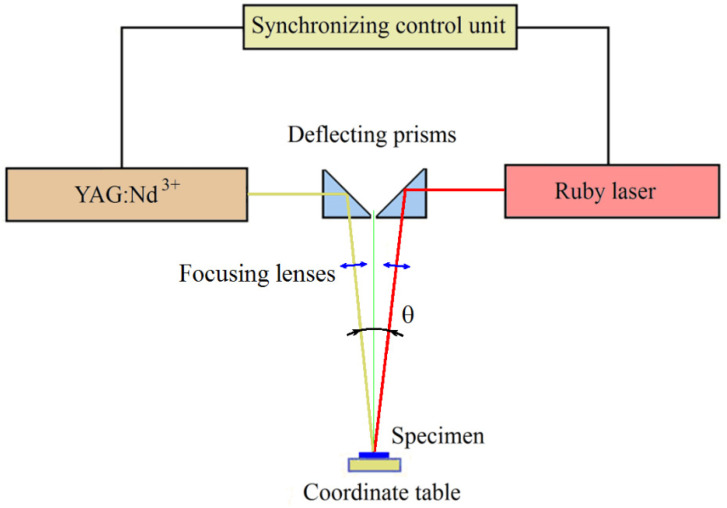
Diagram of a pilot plant for combined LSH and LSP processing of steels.

To conduct research on the effect of combined laser treatment on the mechanical properties and abrasive wear resistance of steels, a pilot plant was developed, the scheme of which is shown in Figure 4. The basis of this installation consisted of two lasers (a pulsed YAG:Nd3+ laser operating in the free-running mode and a ruby laser operating in the Q-switched mode), as well as a synchronized control unit that allows you to vary the energy and amplitude–time radiation modes of each laser and the order of their activation.

The pulsed YAG:Nd3+ laser operating in the free-running mode was used for the surface thermal hardening (LSH) of the studied steels.

The basic parameters of pulsed radiation YAG:Nd3+ lasers are as follows:

- Radiation wavelength—1.06 microns;
- Radiation energy in a pulse—up to 25 J;
- The duration of the radiation pulse is ~5 ms;
- Pulse repetition rate—up to 10 Hz;
- The diameter of the focus spot is 0.7–4 mm.

The ruby laser, operating in the Q-switched mode [24], was intended for shock wave processing (LSP) of the material surface [25]. The main parameters of the ruby laser are as follows:

- Radiation wavelength—0.69 microns;
- Radiation energy in a pulse—up to 1.5 J;
- The duration of the radiation pulse at half–height (FWHM) is 30 ÷ 35 ns;
- Pulse repetition rate—up to 10 Hz;
- The diameter of the focus spot is 0.7–2 mm.

The radiation energy of the YAG:Nd3+ laser and the ruby laser was measured by a specialized solid-state calorimeter, the W-12K-D55-SHC-U.

The amplitude of the compression pulses on the surface of the samples varied in the range from 0.1 to 1 GPa due to the change in the radiation energy in the pulse with a constant pulse duration and the area of the focus spot. Two aspherical lenses with a focal length of 63.5 mm and a diameter of 27.94 mm were used to focus the laser radiation. Aspherical lenses provide a high power density on the workpiece compared to other types of lenses (for example, flat–convex) with equivalent focal lengths. The lenses were mounted on standard mounts for optical elements with the possibility of precise adjustment along three axes (X, Y, and Z). Focusing the laser radiation on the sample surface was carried out by moving the focusing lenses. The angle (θ) in Figure 4 has a value of ~60.

In order to establish the relationship between the pressure and duration of the shock disturbance caused on the surface of the material by pulses of ruby laser radiation and structural and phase changes in the near-surface layer of the metal, measurements of the parameters of laser-induced shock pulses were carried out in this work. For this purpose, a Michelson displacement laser interferometer [26] was used, which has a number of advantages (contactless, inertia-free, noise immunity).

**Figure 5:**
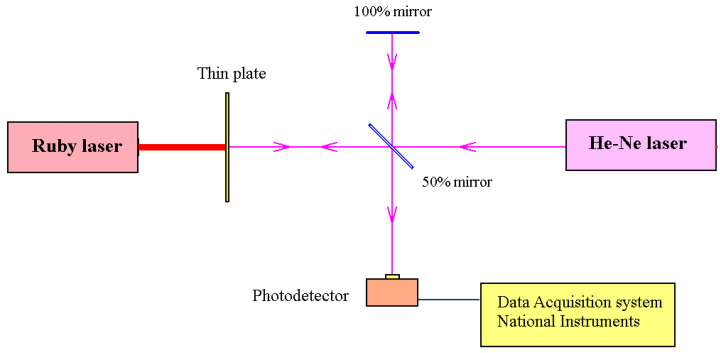
Scheme of the Michelson interferometer.

The main components of the interferometer are the single-mode helium–neon laser OSK-6328-5P OptoSigma (INSCIENCE, Moscow, Russia), 100% and 50% mirrors, the S1336 Hamamatsu photodetector, a thin metal sample with a polished surface, and the National Instruments data acquisition system for recording and processing interferograms (Figure 5).

During calibration experiments, a thin steel plate (0.1 mm thick) on the back side was exposed to a giant monopulse of a ruby laser with a duration of about 30 ns at half height. The front side of the plate was polished to Ra = 0.04 microns and was used as one of the mirrors of the Michelson laser interferometer. A single-mode helium–neon laser with a radiation power of 5 mW was used as a coherent radiation source in the interferometer. The separation of the initial beam into two equal parts was carried out using a translucent mirror: the part of the beam reflected from this mirror was the reference beam, and the part directed at the front surface of the sample plate was the probing beam. When the sample surface is displaced, the frequency of the probing beam reflected from it changes in accordance with the Doppler effect. Reflected, respectively, from the stationary mirror of the interferometer and the moving surface of the sample, the reference and probing rays are mixed on the translucent mirror. The total stream of interfering rays containing the difference frequency is fed to the photodetector and then to the National Instruments data collection system for recording and processing interferograms.

A mechanical compression pulse excited by a laser pulse propagates in the body of a thin plate, and, after a while, reaches its opposite free surface. This pulse is reflected from the free surface by a stretching pulse. As a result, this surface begins to move with a certain velocity, V, the value of which is related to the mass velocity of the particles (u) in the compression wave by the ratio V = 2u (the rule of doubling the velocities). As a result of processing interferograms, the dependence of the displacement (x(t)) of the sample surface on time is obtained. Differentiation of this dependence makes it possible to obtain the desired curve of the change in the velocity of displacement of the surface in time: V = dx/dt.

The mass velocity of the particles in the compression wave can be calculated from the measured surface velocity, u = V/2. Using the acoustic approximation from the law of the conservation of momentum in a compression wave, the load amplitude (σ) is related to the mass velocity (u), the density of the material (p), and the velocity of elastic waves (C) in the plate material by the ratio σ = ρCu. The implementation of tests with different values of the energy of the laser monopulses made it possible to calibrate the radiation of the ruby laser and obtain a correspondence between the radiation energy and the magnitude of the induced pulsed mechanical pressure in the sample. The results of measuring the pressure amplitude of the laser shock wave are given in Table 2.

Next, we will focus on some features of the change in the structural and phase composition of metal alloys under the action of LSH and LSP.

LSH makes it possible to form compressive microstresses in the near-surface layers (0.1–1.0 mm) of the material and significantly grind the grain structure and increase the hardness, wear resistance, fatigue, corrosion, and other operational properties of finished products. This study on the hardening of steels of various compositions has shown that the hardness of the hardened zone often significantly exceeds the hardness obtained during classical furnace heat treatment. The phase and structural transformations that occur when exposed to laser radiation in the near-surface layers of steels are determined by the combination of heating, holding at a fixed temperature, and cooling, i.e., the thermal cycle. Laser thermal hardening usually uses the simplest thermal cycle, consisting of two stages: (1) heating the surface layer during the action of laser radiation and (2) cooling the surface layer due to heat removal deep into the material. Each steel grade has its own thermal cycle that is most favorable from the point of view of obtaining certain specified properties. The thickness of the hardened layer is one of the main characteristics of laser hardening. Its value is currently mainly calculated by the quenching isotherm, and the temperature field in the material is determined by solving the problem of thermal conductivity in various formulations. The parameters of the hardened layer (the depth of the hardened layer and the microhardness in the zone of thermal influence) strongly depend on the degree of dispersion of the real structure affecting the thermal conductivity of the material [27,28].

Laser hardening of carbon and alloy steels for maximum structural hardness is manifested in the formation of a complex structure including fine martensite, residual austenite, and other phase components [29]. The least stable phase is residual austenite, the concentration of which depends both on the initial phase–structural state and chemical composition of the steel, and on the kinetics of the thermal cycle of laser exposure. As a rule, the concentration of residual austenite in the structure of hardened steel increases with an increase in the amount of carbon and alloying elements, as well as with an increase in the cooling rate. Residual austenite has a significant impact on the performance of the modified material. Despite the lack of an unambiguous opinion on the effect of residual austenite on the properties of steel, as a rule, they try to eliminate it and, thereby, ensure the most complete course of martensitic transformation. For the destruction of residual austenite, two methods are usually used: thermal and deformation. The first one is most widely used in technology to regulate the content of residual austenite in processed products. In particular, to reduce the content of residual austenite, high tempering (heating and holding at temperatures above 400–500 °C) or cold treatment is sometimes used, which consists of cooling the steel after quenching to negative temperatures close to the end point of the martensitic transformation. The use of thermal methods after laser heat treatment in order to reduce the content of residual austenite leads to structural changes of a volumetric nature; therefore, along with improving the operational properties of the surface, a decrease in the strength characteristics of the product may occur.

The deformation mechanism of the decay of residual austenite is practically not used in the field. An exception is the method, the meaning of which is the effect of ultrasound on the laser-hardened surface of steel. As a result of ultrasonic exposure, characterized by a rather high deformation rate of ~10^3^ 1/s, the content of residual austenite decreases and an ultrafine-grained equilibrium structure is created without peak local residual stresses [30].

LSP exposure to metals and alloys is accompanied by various mechanical and physico-chemical processes, including residual hardening. Laser-induced shock waves of non-destructive amplitude, dissipating their energy during propagation inside metals, lead to irreversible changes in their structure. There is no single mechanism of hardening under the influence of shock waves for metals and alloys that differ in physical and mechanical properties, crystal structure, and chemical composition. Often, the degree of hardening is determined by the amplitude of the shock wave pressure, and in other cases by the amount of deformation behind the front, the shape and duration of the compression pulse, etc. However, the degree of hardening always depends on the amount of distortion remaining in the crystal lattice of the metal after the passage of the shock wave. Distortions of the structure can occur at the submicro, micro, and macro levels.

A distinctive feature of shock loading is an extremely high deformation rate due to the short duration of the pressure pulse (less than 0.1 microseconds).

Dislocations, dislocation cells, deformation, and twin packing defects, as well as other flat dislocation clusters, twins, point defects, and their clusters, make a significant contribution to the microstructure formed during pulsed loading. The formation and density of these crystal defects depend on the applied pressure. Under shock loading, the concentration of deformation vacancies increases. With increasing pressure, the dislocation density increases proportionally to the square root of the pressure at the shock wave front.

For Fe-C alloys with LSH treatment, the depth of the hardened zone and the magnitude of microhardness depend on the structural state of the material, the optical properties of the surface (the absorption coefficient of laser radiation), and its thermal properties (thermal conductivity and thermal conductivity). In turn, LSP treatment leads to noticeable structural and phase changes in the surface layer, characteristic of shock wave loading [3]. Based on this, it was suggested that the combination of LSH and LSP treatments can lead to an increase in LSH parameters and to an increase in the abrasive wear resistance and strength properties of steels.

**Figure 6:**
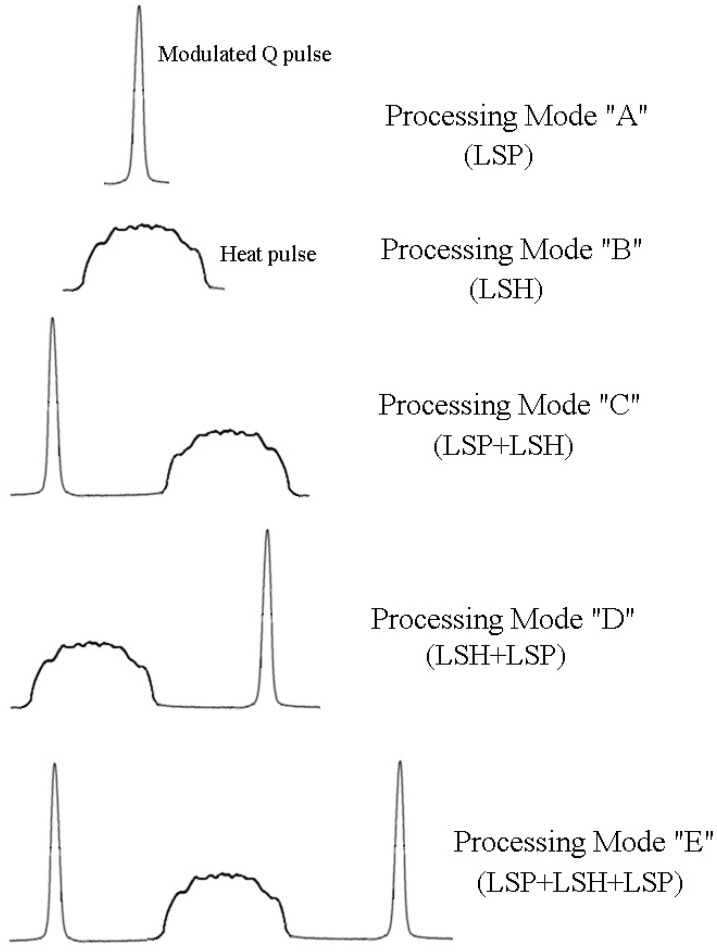
Operating modes of the laser installation.

Next, we will consider the proposed and implemented modes of operation of the installation for combined laser surface treatment, schematically shown in Figure 6.

Mode “A”—LSP: only the ruby laser works in the Q-switched mode for shock wave processing of the sample surface.

Mode “B”—LSH: the YAG:Nd3+ laser is used in free-running mode for surface thermal hardening of the sample.

Mode “C”—LSP + LSH: first, the surface of the sample is treated with a ruby laser in the Q-switched mode (preliminary shock wave treatment to saturate the surface layer of the metal with structural defects, which leads to a more efficient phase transition during laser quenching); then, exposure to pulsed radiation is performed with the YAG:Nd3+ laser in free-running mode for thermal hardening of the surface layer.

Mode “D”—LSH + LSP: initially, the surface is exposed to the YAG:Nd3+ laser in the free-running mode (thermal hardening); then, after a pause, which is necessary to cool the laser exposure zone to room temperature (~12 ms), the surface is treated with a ruby laser in the Q-switched mode (shock wave treatment to change the phase composition of the hardened layer).

Mode “E”—LSP + LSH + LSP: First, the surface of the sample is treated with a ruby laser in the Q-switched mode (preliminary LSP) to saturate the surface layer of the metal with structural defects, which leads to a more efficient phase transition during laser quenching. Then, the effect is produced by pulsed radiation from the YAG:Nd3+ laser in the free-running mode, which leads to thermal hardening (LSH) of the surface layer. Then, as in mode D, after a pause of ~12 ms, the surface is re-treated with a ruby laser in the Q-switched mode (LSP) to change the phase composition of the hardened layer.

All three types of samples of the three studied steel grades described in Section 2 were subjected to these laser surface treatment modes.

### 3.2. Methods of Studying the Wear Resistance of Steels

The main loss of material during the operation of steel parts of machines and mechanisms is associated with wear. Abrasive wear can be defined as “wear caused by solid particles or solid protrusions colliding with a solid surface and moving along it” [31]. It is believed that the share of abrasive wear in the total wear is 20% [32]. Wear characteristics are influenced by many factors that are sometimes difficult to quantify. Hardness, as one of the most important characteristics of alloys, often affects the wear rate, including during abrasive wear [33].

There are many types of abrasive tests, some of which are standardized. One of the most commonly used types of testing is the abrasion of the test surface with dry sand carried away by a rubber disc, which is designated as DSRW (dry sand, rubber wheel).

**Figure 7:**
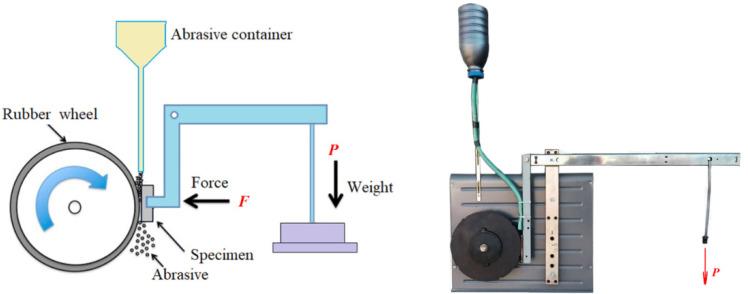
Diagram (**left**) and appearance (**right**) of the Brinell–Haworth machine.

The abrasive wear resistance of the surfaces of samples subjected to LSH and LSP treatment in their various combinations was tested using the Brinell–Haworth wear unit (Figure 7) [34,35]. The test sample is pressed against a rotating rubber disc with a given load (F). A loose abrasive is fed into the abrasion zone from the hopper. When the wheel rotates, the abrasive grains are captured by the rubber surface and slip relative to the metal sample, exposing it to wear (three-body abrasion). At the moment of contact, the rubber covers the abrasive particle and creates lateral supporting forces that limit its ability to turn in contact with the metal.

The studied flat samples (Figure 1) with a size of 40 × 10 × 4 mm made of steels 20, 45, and U8 were pressed against a counterbody rotating at a constant speed with force F.

The tests were carried out under the following conditions:

(a) Quartz sand with a fraction of 0.2–0.3 mm was fed from a hopper with a calibrated hole into the gap between the rubber disc and the sample;
(b) The hole was wiped with a rotating rubber disc on a flat sample of the tested metal at constant load (F) and wheel rotation speed;
(c) The diameter of the rubber disc was 200 mm, with a width of 12 mm and a rotation speed of 200 rpm;
(d) The total duration of the sample test was 20 min, which corresponds to 4000 revolutions of the disk or 2513 m of the total friction path;
(e) The load on the sample was 150 N.

After every 4 min, the rotation of the disk stopped and the sample was removed from the installation, washed with gasoline, and weighed on analytical scales.

This set of procedures was repeated at least five times and the results were averaged, and then a statistical analysis was carried out to determine confidence intervals.

Wear resistance was estimated by the weight loss of the sample for a fixed test time, as an average value based on the test results of five samples. The samples were weighed on VIBRA HT/HTR 220TE electronic scales with a measurement accuracy of 0.0001 g.

### 3.3. Method of Investigation of Microhardness of Samples

The microhardness of the hardened layers was measured on specially prepared transverse micro-grinders with a PMT-3M microhardness meter with a load of 0.5 N (50 g). The load application time was 10 s. Using the obtained values of microhardness, its distribution over the depth of the hardened layer was obtained for each treatment mode of the studied steels.

### 3.4. Methods of Investigation of Mechanical Properties of Steels

In order to fully characterize the considered combinations of laser shock wave and laser thermal effects, in this work, the influence of various modes of laser thermal power treatment on the mechanical properties of the studied steels during static and dynamic tests was investigated.

Tensile tests are the main and most common way to determine the mechanical characteristics of materials. The tensile test gives the most complete picture of the mechanical properties of the metal, as it provides a unique opportunity to implement a homogeneous stress state, in which it is not necessary to resort to additional hypotheses about the deformation of the sample. In addition, tensile stresses are the most dangerous in real structures and are most often responsible for destruction.

### 3.5. Methods of Investigation of Mechanical Properties of Steels under Static Loads

The LR5KPlus universal testing machine was used to perform tensile tests under static loads. The maximum axial force was 5 kN. The tests were carried out on flat samples made of steel 20 and steel 45 (Figure 2), both in the initial state and after various combinations of LSH and LSP.

### 3.6. Methods of Investigation of Mechanical Properties of Steels under Dynamic Loads

**Figure 8:**
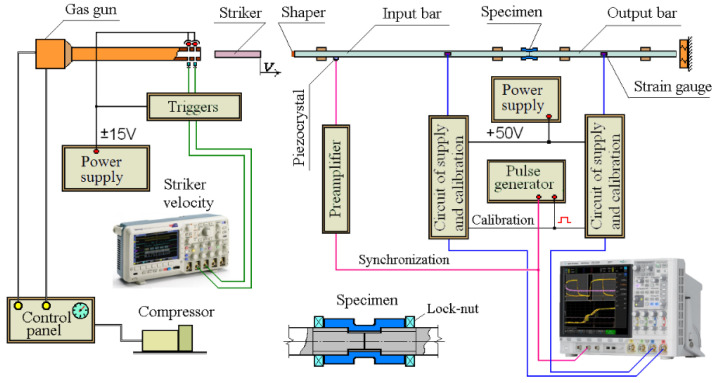
Diagram of an experimental setup for dynamic tensile testing of tubular samples.

To study the effect of various modes of combined laser processing on dynamic properties, tubular samples (Figure 3) of structural steels 20 and 45 were tested for uniaxial tension on an installation implementing the Kolsky technique with a Hopkinson split bar [36]. The scheme of the installation for dynamic loading of samples with a tensile load is shown in Figure 8. A modification of the Kolsky technique proposed by Nicholas [37] was used to form a tensile load in the Hopkinson split bar system. Figure 8 also shows the attachment of the tubular sample to the ends of the measuring bars.

**Figure 9:**
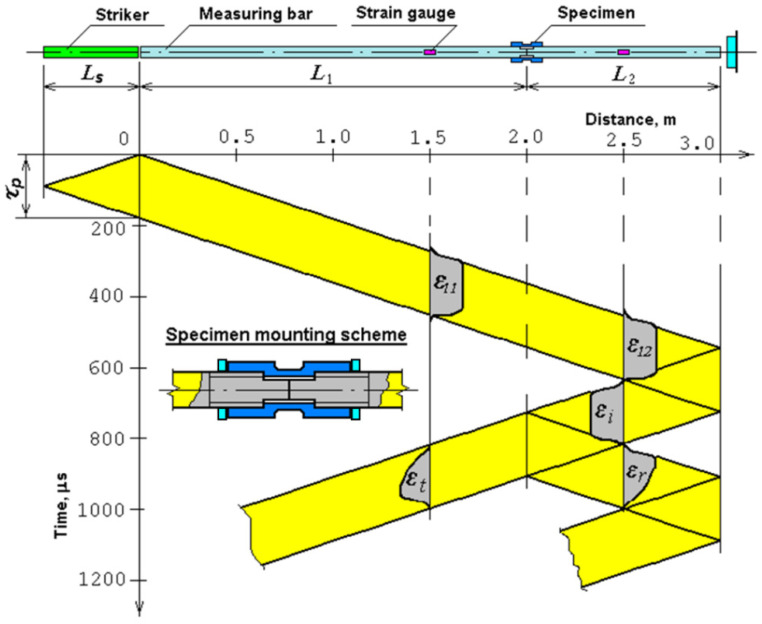
Lagrangian x-t diagram during tensile tests.

In this scheme, the sample is loaded by a stretching wave, which is formed after the compression wave is reflected from the free end of the support bar. Figure 9 shows the Lagrangian diagram of elastic wave propagation in the Hopkinson split bar system using the Nicholas modification. The tubular sample is screwed onto the ends of the bars with nuts. The specially treated ends of the bars, from which part of the thread has been removed, are joined tightly inside the tubular sample, so that a small cylindrical gap is formed between the central smooth zone of the inner surface of the sample and the lateral surface of the ends of the bars, allowing the incident compression wave to pass freely into the second bar.

The essence of the method is as follows. A longitudinal compression pulse is applied to the left end of the first dimensional bar and excites an elastic one-dimensional compression wave, ε_11_(*t*), in it. This initial pulse freely passes through the joined ends of the measuring bars, without causing plastic deformation of the sample, into the second bar by a wave of ε_12_(*t*) and, reaching the free end of this bar, is reflected by a stretching wave. This stretching pulse is the initial incident wave, ε_i_(*t*), for the stretching cycle of the sample. From the moment of reflection and formation of the stretching pulse from the free end of the bar, the experimental scheme is similar to the compression test scheme. The stretching impulse, having reached the sample, partially passes through it into the first bar by the stretching wave, ε_t_(*t*), and is partially reflected back into the second bar by the compression wave, ε_r_(*t*). The sample undergoes plastic deformation. Using the basic dependences of the Kolsky method, it is possible to determine the processes of development in time of the stress (σ_s_(*t*)), strain (ε_s_(*t*)), and strain rate (έ_s_(*t*)) of the sample and then construct the curve, σ_s_(ε_s_).

### 3.7. Method of Evaluating the Decay of Residual Austenite after the Action of Laser-Induced Shock Waves (LSP Treatment)

X-ray structural studies of the hardened layers of the studied steel grades after modes B and C, where the treatment was completed by laser thermal exposure (LSH) with a power flux density of P~3.5·10^4^ W/cm^2^, showed a high content of residual austenite in the hardened steel structure: about three percent for steel 20, about eight percent for steel 45, and twenty-eight percent for steel U8.

Residual austenite is an undesirable phase in the hardened surface layer, negatively affecting the mechanical and tribological properties. The surface of the samples hardened by laser radiation was exposed to intense pressure pulses, which were excited by pulses of ruby laser radiation (radiation parameters: pulse energy up to 1.5 J, pulse duration τ = 30 ns). The amplitude of the shock wave was controlled by changing the radiation energy of the laser pulse. At the same time, the pulse duration and the diameter of the focus spot remained unchanged.

The dependence of the change in the content of residual austenite on the number of laser irradiation pulses was obtained for a fixed value of the irradiation pulse energy, E = 1 J, which corresponded to the value of the amplitude of the compression pulse on the sample surface, P = 0.65 GPa. In the experiments, the number of pressure pulses varied from 5 to 30; after every 5 pulses, diffractograms were taken from the treated surfaces of the samples, according to which the amount of residual austenite was calculated. To obtain more reliable results, 3 repeated diffraction patterns were taken in each case and the amount of retained austenite was calculated for each diffractogram. The resulting triplets of results in each section were averaged, statistical analysis was carried out, and confidence intervals were determined.

The amount of residual austenite was determined by pairs of lines (110)α–(111)γ and (211)α–(200)γ, while the calculation was carried out according to the well-known formula, where Iα and Iγ are integral intensities and K is the coefficient. For a pair of lines (110)α–(111)γ, K = 0.77 [38]. When calculating along the lines (211)α–(200), the latter was normalized to an intensity of 100% and then the calculation was performed using the same coefficient.

## 4. Results and Discussion

### 4.1. Investigation of the Decay of Residual Austenite under the Action of Laser-Induced Shock Waves (LSP Treatment)

**Figure 10:**
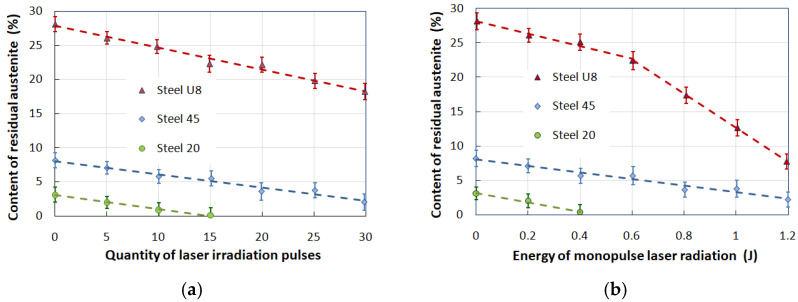
Dependence of the residual austenite content in the hardened steel structure on the number of fixed pressure pulses (**a**) and on the radiation energy of the laser monopulse (**b**).

The measurement results showed that with an increase in the number of pressure pulses acting on the structure of laser-hardened steel, the amount of residual austenite decreases linearly. This fact indicates (for a given range of pressure pulse amplitudes) the cumulative nature of the decay process of residual austenite. The dependence of the residual austenite content in the structure of hardened steel on the number of fixed pressure pulses (P = 0.65 GPa) is shown in Figure 10a. For low-carbon steel 20, after 15 pulses of laser irradiation, there is no residual austenite.

To study the regularity of the change in the amount of residual austenite depending on the energy of the ruby laser monopulses, pre-hardened samples of the studied steels were treated with LSP compression pulses, the amplitude of which varied from 0.12 to 0.8 GPa, which corresponded to a change in the energy of the radiation pulse from 0.2 to 1.2 J. After exposure to nanosecond pulses of radiation at each energy value, diffractograms were taken from the samples and the number of austenitic and martensitic phases was calculated. Studies have shown that noticeable changes in the content of residual austenite occur when the radiation energy and, accordingly, the amplitude of the compression pulse exceed a certain threshold value (for steel U8). No threshold changes were observed for steel 45: the dependence of the amount of residual austenite on the radiation energy is linear (Figure 10b). One of the reasons for the absence of a threshold value for steel 45 in this graph may be the limited range of pressures used in experiments.

### 4.2. Investigation of the Effect of Combined Laser Exposure on the Parameters of Laser Hardening (Depth and Microhardness of Hardened Zones)

**Figure 11:**
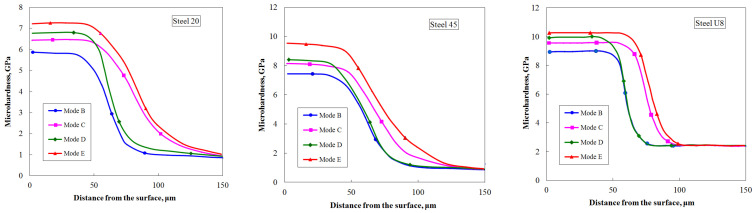
Dependences of the microhardness distribution over the depth of the hardened layer for the studied steels after various combinations of laser treatment.

The results of measuring the microhardness of the surface-hardened layers of the studied steels, shown in Figure 11, showed that the nature of the increase in microhardness is the same for all steels and depends on the laser treatment modes. Thus, the maximum increase in microhardness compared to traditional laser hardening (mode B) is observed after processing according to mode E—by 22% for steel 20, by 27% for steel 45, and by 13% for steel U8. In addition, along with the increase in microhardness, there is a noticeable increase in the depth of the hardened layer. For example, after processing according to mode E compared to mode B, an increase in the depth of the hardened layer was recorded—by 1.53 times for steel 20, by 1.41 times for steel 45, and by 1.29 times for steel U8.

### 4.3. Investigation of Mechanical Properties of Steels after Combined Laser Exposure

The creation of a modified surface layer after combined laser exposure (as well as after traditional laser hardening) affects the mechanical behavior of Fe-C alloys under static and dynamic loads. The structure of the hardened layers, which affects the mechanical properties, is characterized by high dispersion and high hardness.

Tensile tests are the main and most common way to determine the mechanical characteristics of materials, since they give the most complete picture of the mechanical properties of the metal. In addition, tensile stresses are the most dangerous in real structures and are most often responsible for destruction.

#### 4.3.1. Results of Static Tests

Table 3 shows the average values of mechanical characteristics obtained during static tensile tests of steel samples 20 and 45 after various processing modes.

The maximum increase in the values of σ_0.2_ and σ_B_ was observed in samples treated according to modes “C” and “E”, which is associated with an increase in the volume fraction of the hardened metal and the high values of the microhardness of the hardened layer. All processing modes are characterized by a sharp drop in plasticity. Rather low values of plasticity of structural steels after processing according to modes “B” and “C” are associated with the occurrence of cracks on the treated surface that appear during stretching, the development of which is perpendicular to the direction of deformation. The cracks start from the center of the laser exposure zone and extend to the edge of the flat sample. The formation of cracks in the laser exposure zone is favored by large thermal stresses resulting from laser treatment. The impact of the shock pulse after laser quenching (modes “D” and “E”) slightly reduce the stress level, which leads to a slight increase in plasticity.

The nature of the change in the mechanical properties of steel 45 is generally similar to the changes in the mechanical properties of steel 20. It should be noted that for steel 45, the most effective is processing according to modes “D” and “E”, after which there is an increase in the values of σ_0.2_ and σ_E_ with a simultaneous increase in plasticity values compared to modes “B” and “C”. Cracks in the laser-hardened layer are formed at the stage of fluidity and deformation hardening. No cracks were found on samples loaded up to σ_E_. Analysis of the nature of the destruction of samples made of steels 20 and 45 after all the treatment modes implemented shows that the fracture surface passes through the laser tempering zones (in places where the zones of thermal influence overlap), which have a reduced microhardness compared to hardened surface areas. The conducted studies have shown that the increase in the mechanical characteristics of steels after combined laser treatment is associated with the formation of a hardened layer with greater depth and hardness (modes “C” and “E”) than after traditional laser hardening. The most preferable (from the point of view of the mechanical properties of steels) processing mode for steels 20 and 45 of those considered is mode “E”, in which, along with high values of σ_0.2_ and σ_E_, the value (magnitude, level, degree, index) of plasticity also increases.

After laser treatment, a hardened layer of various depths and hardness is formed on the surface of the samples. The distribution of microhardness within the hardening zones is fairly uniform. Table 4 shows the average values of the depth of the hardened zones and the average values of microhardness after the implemented laser treatment modes.

#### 4.3.2. Results of Dynamic Tests

The study of the behavior of Fe-C alloys subjected to laser surface hardening during high-speed deformation is of great scientific and practical interest in connection with the development of a number of areas of new technology and processing technologies.

**Figure 12:**
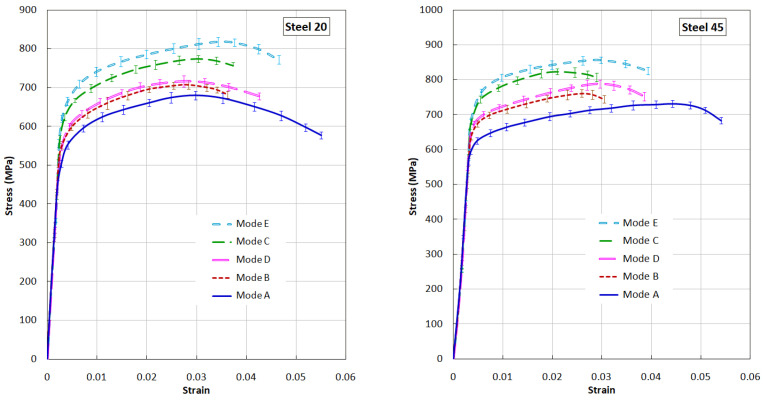
Dynamic diagrams of steels 20 and 45 under tension after various modes of surface laser treatment.

Using the Kolsky method described above, dynamic tests for uniaxial tension of tubular specimens (Figure 3) were carried out and dynamic deformation diagrams were obtained at a strain rate of ~10^3^ s^−1^. To increase the reliability of the results, a standard statistical analysis of the stress–strain diagrams obtained as a result of three repeated experiments under the same conditions was carried out. Averaging of the stress–time and strain–time curves was carried out on a common time axis using Excel with the determination of confidence intervals. After preliminary synchronization, for each time slice, on the parametric “time–strain” and “time–stress” dependencies, the average values of the corresponding quantities and confidence intervals are determined, containing the true value of the random variable with a given probability. The analysis of the obtained results (Figure 12) showed changes in the mechanical properties of steels 20 and 45 after various modes of surface laser treatment. These changes are related to the depth and structure of the hardened layers formed after various processing modes.

### 4.4. Abrasive Wear Tests

The results of measuring the wear intensity of samples that underwent combined laser–shock wave treatment (modes “C” and “D”) were compared with similar values obtained on steel samples that underwent traditional laser hardening (mode “B”).

**Figure 13:**
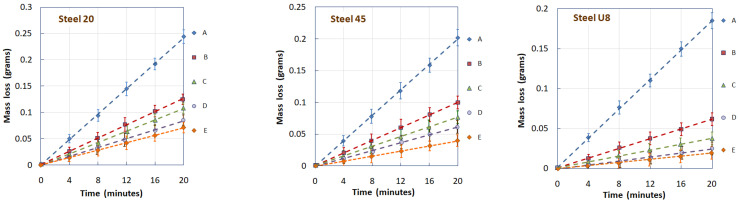
Dependence of the mass loss of the sample on the test time after various laser treatment modes for steels 20, 45, and U8.

Figure 13 shows the dependences of the mass loss of samples from steels 20, 45, and U8 on the test time after various types of processing. All dependencies are shown with confidence intervals determined by standard methods of statistical analysis.

**Figure 14:**
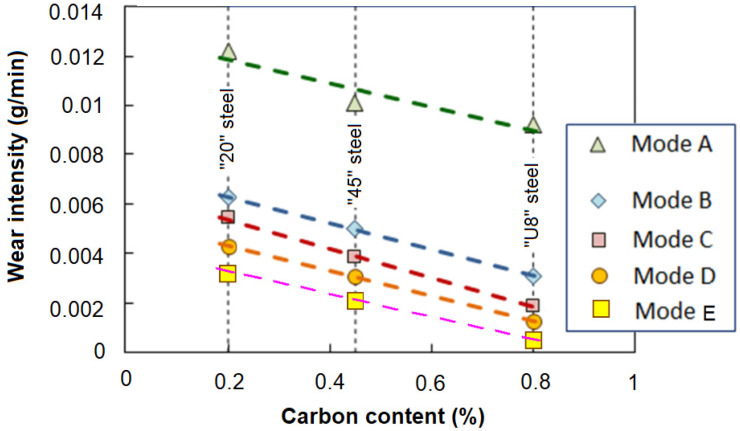
The wear intensity of the samples of the studied steels.

The conducted studies have shown that in LSP mode “C”, the treatment in the first stage contributes to the saturation of the surface layer of the metal with structural defects, which leads to a more efficient phase transition during subsequent LSH. On the other hand, treatment with laser-induced shock waves of an already hardened surface (mode “D”) leads to a decrease in the content of residual austenite in it. As a result of processing in both modes “C” and “D”, the abrasive wear resistance of the studied steels increases significantly compared to the wear resistance of the surface hardened by traditional laser quenching technology (mode “B”). However, in the considered treatment combinations, LSP treatment following LSH (mode “D”) is more effective than LSP before LSH (mode “C”), since a decrease in the concentration of residual austenite in the structure of hardened steel under the condition of abrasive wear is a more significant factor in increasing wear resistance than the formation of fine martensite when quenching a structure saturated with defects. The evidence of this is shown in Figure 14, where for three grades of steels with different carbon contents, a change in the wear intensity of samples that underwent various surface laser treatment options is shown.

For a comparative evaluation of the results obtained, the following parameters were used: the wear rate (I = Δm/Δt) and the relative wear rate (I_t_/I_ut_), where Δm is the loss of mass during wear; Δt is the test time; I_t_ is the wear rate of samples treated with laser thermal or combined methods; and I_ut_ is the wear rate of untreated samples.

Thus, the conducted studies have shown that the combination of laser shock wave (LSP) and laser thermal effects (LSH) is an effective type of modification of the surface of carbon steels to increase their abrasive wear resistance.

## 5. Conclusions

The conducted studies have shown that the use of combined laser treatment modes based on a combination of laser pulses of Q-switched mode and free-running mode in different sequences leads to an increase in the abrasive wear resistance of the studied steels compared with traditional laser hardening. It is established that in the considered combinations, shock wave treatment following laser quenching is more effective than shock wave treatment before laser quenching. This suggests that a change in the phase composition in the structure of hardened steel during shock wave processing is a more significant factor in increasing wear resistance than the formation of fine martensite when hardening a structure saturated with defects. The considered modes of combined laser shock wave and thermal effects can be used to increase the service life of machine parts and mechanisms operating under conditions of abrasive wear.

It has been experimentally established that laser combined treatment increases the strength and lowers the plastic properties of structural steels under uniaxial tensile conditions under static (ε~10^−3^ s^−1^) and dynamic (ε~10^3^ s^−1^) loading, and the degree of change in mechanical properties depends on the depth and structural–phase composition of the hardened layer. These modes of combined laser exposure can be used to increase the specific strength of structural elements.

## Figures and Tables

**Table 1:** Chemical composition (%) of the studied steels.

Steel Grade	C	Si	Mn	Ni	S	P	Cr	Cu	As
“20” steel	0.17–0.24	0.17–0.37	0.35–0.65	≤0.3	≤0.04	≤0.035	≤0.25	≤0.3	≤0.08
“45” steel	0.42–0.5	0.17–0.37	0.5–0.8	≤0.3	≤0.04	≤0.035	≤0.25	≤0.3	≤0.08
“U8” steel	0.75–0.84	0.17–0.33	0.17–0.33	≤0.25	≤0.028	≤0.03	≤0.2	≤0.25	N/S

**Table 2:** Dependence of the laser shock wave pressure (P) on the energy (E) of a monopulse of ruby laser radiation at fixed values of the radiation pulse duration (FWHM~30 ÷ 35 ns) and focusing spot area ~1.5 mm^2^.

E, J	0.4	0.6	0.8	1.0	1.2	1.4
P, GPa	0.2	0.36	0.45	0.65	0.8	0.93

**Table 3:** Average values of static mechanical characteristics of the studied steels obtained after various laser treatment modes.

Processing Mode	h (μm)	H_μ_ (GPa)	σ_0.2_ (MPa)	σ_B_ (MPa)	δ (%)
“20” steel					
Without processing		1.1	280	440	34
Mode A		1.1	280	440	34
Mode B	75	6.0	340	520	5.8
Mode C	115	6.5	360	540	5.6
Mode D	75	6.0	340	530	6.2
Mode E	115	6.6	380	560	6.0
“45” steel					
Without processing		1.8	320	550	13
Mode A		1.8	320	550	13
Mode B	85	7.2	380	620	2.8
Mode C	120	7.8	400	630	2.4
Mode D	85	7.3	380	630	3.4
Mode E	120	7.9	410	640	3.0

**Table 4:** Average depth values and average microhardness values of the hardened zones of steels “20” and “45” after treatment with different combinations of laser thermal and shock wave effects.

Material	Parameter	Mode B	Mode C	Mode D	Mode E
“20” steel	h (μm)	120	140	120	140
	Hμ (GPa)	6	6.5	6.6	6.6
“45” steel	h (μm)	100	120	100	120
	Hμ (GPa)	7.2	8	7.3	8.1

## Data Availability

Data are contained within the article.
